# Soluble Interleukin-6 Receptor Regulates Interleukin-6-Dependent Vascular Remodeling in Long-Distance Runners

**DOI:** 10.3389/fphys.2021.722528

**Published:** 2021-10-11

**Authors:** Paulina Villar-Fincheira, Aaron J. Paredes, Tomás Hernández-Díaz, Ignacio Norambuena-Soto, Nicole Cancino-Arenas, Fernanda Sanhueza-Olivares, Felipe Contreras-Briceño, Jorge Mandiola, Nicole Bruneau, Lorena García, María Paz Ocaranza, Rodrigo Troncoso, Luigi Gabrielli, Mario Chiong

**Affiliations:** ^1^Advanced Center for Chronic Diseases (ACCDiS) & CEMC, Faculty of Chemical and Pharmaceutical Sciences, Universidad de Chile, Santiago, Chile; ^2^Advanced Center for Chronic Diseases (ACCDiS), Faculty of Medicine, Pontificia Universidad Católica de Chile, Santiago, Chile; ^3^Laboratory of Exercise Physiology, Department Health of Science, Faculty of Medicine, Pontificia Universidad Católica de Chile, Santiago, Chile; ^4^Center of New Drugs for Hypertension, Universidad de Chile & Pontificia Universidad Católica de Chile, Santiago, Chile; ^5^Laboratorio de Investigación en Nutrición y Actividad Física (LABINAF), Instituto de Nutrición y Tecnología de los Alimentos (INTA), Universidad de Chile, Santiago, Chile

**Keywords:** interleukin-6, soluble interleukin-6 receptor, vascular smooth muscle cell, runners, exercise

## Abstract

Little is known about the effects of training load on exercise-induced plasma increase of interleukin-6 (IL-6) and soluble IL-6 receptor (sIL-6R) and their relationship with vascular remodeling. We sought to evaluate the role of sIL 6R as a regulator of IL-6-induced vascular remodeling. Forty-four male marathon runners were recruited and allocated into two groups: low-training (LT, <100 km/week) and high-training (HT, ≥100 km/week), 22 athletes per group. Twenty-one sedentary participants were used as reference. IL-6, sIL-6R and sgp130 levels were measured in plasma samples obtained before and immediately after finishing a marathon (42.2-km). Aortic diameter was measured by echocardiography. The inhibitory effect of sIL-6R on IL-6-induced VSMC migration was assessed using cultured A7r5 VSMCs. Basal plasma IL-6 and sIL-6R levels were similar among sedentary and athlete groups. Plasma IL-6 and sIL-6R levels were elevated after the marathon, and HT athletes had higher post-race plasma sIL-6R, but not IL-6, level than LT athletes. No changes in sgp130 plasma levels were found in LT and HT groups before and after running the marathon. Athletes had a more dilated ascending aorta and aortic root than sedentary participants with no differences between HT and LT athletes. However, a positive correlation between ascending aorta diameter and plasma IL-6 levels corrected by training load and years of training was observed. IL-6 could be responsible for aorta dilation because IL-6 stimulated VSMC migration *in vitro*, an effect that is inhibited by sIL-6R. However, IL-6 did not modify cell proliferation, collagen type I and contractile protein of VSMC. Our results suggest that exercise induces vascular remodeling. A possible association with IL-6 is proposed. Because sIL-6R inhibits IL-6-induced VSMC migration, a possible mechanism to regulate IL-6-dependent VSMC migration is also proposed.

## Introduction

Moderate exercise is considered an essential element of maintaining cardiovascular health (Morris et al., 1953). However, repetitive and strenuous exercise in some predisposed athletes also produces marked and likely deleterious changes in cardiovascular morphology and function (Trivax and McCullough, 2012). Training can stimulate the formation of new capillaries by angiogenesis and increase the size of the conduit artery by arteriogenesis (Green and Smith, 2018). This physiological vascular remodeling, known as “athlete’s artery,” increases blood flow to skeletal muscles and other organs to fulfill the nutrient and oxygen requirements of athletes (Green and Smith, 2018). However, excessive or unregulated vascular remodeling could trigger vascular diseases (Lyle and Taylor, 2019).

Myokines are synthesized and released from contracting skeletal muscle cells (Gorgens et al., 2015). Myokines have autocrine, paracrine and endocrine effects on organs, such as adipose tissue, liver, and bone (Gorgens et al., 2015). The most well-known myokine is interleukin-6 (IL-6) (Gorgens et al., 2015). Plasma IL-6 levels increases in response to a variety of acute exercise types (Catoire and Kersten, 2015). The magnitude this increase depends on exercise type, duration and intensity, as well as the amount of muscle mass engaged (Catoire and Kersten, 2015). In skeletal muscle, IL-6 induces hypertrophy (Serrano et al., 2008) and increases insulin sensitivity and fatty acid oxidation (Catoire and Kersten, 2015). Because IL-6 also stimulates VSMC proliferation and migration (Morimoto et al., 1991; Wang and Newman, 2003), reduces VSMC contractility (Ohkawa et al., 1994) and induces production of matrix metalloproteinases (MMP)-9 and MMP-1 (Zhu et al., 2000), it has been proposed that IL-6 could be responsible for exercise-induced vascular remodeling.

Classically, IL-6 binds to the plasma membrane-associated IL-6 receptor (IL-6R). The IL-6/IL-6R complex then associates with gp130, inducing homodimerization and initiation of signaling (Varghese et al., 2002). A soluble form of IL-6R (sIL-6R) has been described in the blood (Wolf et al., 2016). IL-6 can bind to the soluble receptor forming an IL-6/sIL-6R complex (Rosa Neto et al., 2009). The gp130 is expressed by all cells in the body, whereas membrane-bound IL-6R is mainly expressed by hepatocytes and some inflammatory cells (Taga and Kishimoto, 1997; Schmiedel et al., 2018; Monaco et al., 2019). The soluble complex sIL-6R/IL-6 can bind and stimulate cells which only express gp130 (Wolf et al., 2016). The gp130 glycoprotein is also present in a soluble form (sgp130). The sgp130 interacts and inhibits the IL-6/sIL-6R complex, without interfering with the classical IL-6 signaling (Demyanets et al., 2012; Wolf et al., 2016).

The primary aim of this study was to determine the effect of the training load on plasma IL-6, sIL-6R and sgp130 levels in long-distance runners after completion of an intense and prolonged exercise (marathon). The secondary objective was to explore the possible association of IL-6 and sIL-6 on vascular remodeling triggered by exercise.

## Materials and Methods

### Participants

Forty-four male recreational long-distance runners were recruited previously to a marathon race (Santiago, 42.2 km). The participants were included 16 weeks before the competition, in the training period called “optimal phase,” where the volume of training is increased by running more distance by week. The inclusion criteria were: (i) age between 18 and 50 years to minimize possible cardiovascular event linked to competition, (ii) participation in three or more completed marathons previously in the last five years, (iii) recreational status to obtain a more diverse sample. The exclusion criteria were: (i) presence of any morbidity or disease that alter plasma levels of IL-6 (i.e. arterial hypertension, dyslipidemia, insulin resistance, smoking or alcohol consumption habit, renal or liver dysfunction, neoplasia, chronic respiratory, and cardiac diseases); and (ii) use of ani-hypertensive, anorexic, anti-depressant, and/or antibiotics medication. Athletes were divided into two groups according to pre-marathon training load during the 16 weeks before the day of the marathon: high-training group (HT, ≥100 km by week) (*n* = 22) and low-training group (LT, <100 km) (*n* = 22). The 100 km per week cut-off point was based on protocols from our institution, which recommend that high-performance amateur and professional athletes train at least 100 km per week during the period leading up to a marathon race. In addition, a control group of healthy and non-active subjects (CT, *n* = 21) was included. The study was approved by the Ethics Committee of Pontificia Universidad Católica de Chile, in observance of the Declaration of Helsinki on experimentation in humans’ beings (project No 16082603). Written informed consents were obtained from the subjects prior to any procedure.

### Hydration During Exercise

All participants completed the marathon race and hydrated freely using all 12 hydration points (ubicated at 5, 10, 15, 18, 21, 24, 27, 30, 33, 36, 39, and 42 kilometers). The group HT drank 1318 ± 231 mL, while LT drank 1338 ± 340 mL (*p* = 0.89).

### Echocardiography

A transthoracic echocardiography (TTE) study was performed one week before the marathon using a Vivid I portable equipment (GE, Healthcare, Horton, Norway) with a 1.5/3.5 MHz cardiac transducer, according to American Society of Echocardiography guidelines (Lang et al., 2015) to determine the aortic root and ascending aorta diameter (line parasternal long-axis view). Briefly, the aortic root diameter at the sinuses of Valsalva and the ascending aorta diameter were measured from inner edge to inner edge at end-diastole, in a strictly perpendicular plane to that of the long axis of the aorta using the L-L convention. Image quality was optimized to obtain at least 60 frames per second and digitally stored for later analysis using EchoPAC BT 12 software (GE Healthcare, Horton, Norway). Each value of aortic root and ascending aorta diameters was the average of 5 measurements obtained from different cycles, and they were normalized by body surface area before comparison. One trained, single-blinded trained echocardiographer performed the analysis.

### IL-6, sIL-6R, and sgp130 Measurements

Basal venous blood samples were obtained one-week prior to the marathon. Post-exercise venous blood samples were obtained at the finish line within 5 min of the end of the marathon. IL-6, sIL-6R and sgp130 were determined using ELISA kits (ab178013, ab46029, and ab46135, Abcam, Cambridge, United Kingdom, respectively). Samples were analyzed in duplicates. However, if the differences between both samples were higher than 20%, the samples were re-analyzed

### Cell Culture

Direct effects of IL-6 and sIL-6R on VSMCs were assessed in cultured cells. The A7r5 embryonic rat aorta-derived cell line was purchased from the American Type Culture Collection (ATCC, CRL-1444). Cells were cultured in Dulbecco’s modified Eagle’s medium (DMEM) supplemented with 10% fetal bovine serum (FBS) and antibiotics (penicillin and streptomycin) at 37°C, with 95% air and 5% CO_2_. Prior to stimulation, A7r5 VSMCs were partially serum-deprived overnight with DMEM 2% FBS. Experiments were performed between passages 4 and 10.

### Migration and Proliferation Assays

The wound healing assay was performed in A7r5 VSMCs seeded in 60 mm culture plates at maximum confluence cultured for 24 h in DMEM 2% FBS medium as described (Garcia-Miguel et al., 2018; Norambuena-Soto et al., 2020). Cells were treated with IL-6 (300 ng/mL, ab218726, Abcam, Cambridge, United Kingdom), sIL-6R (300 ng/mL, ab167742, Abcam, Cambridge, United Kingdom), IL6 + IL6R or PDGF-BB (20 ng/mL, #521225, Millipore Sigma, Burlington, MA, United States) for 24 h. Migration was expressed as a percentage of wound closure compared to initial cell-free area at time 0 h.

A transwell assay was performed using 8 μm pore Boyden chambers in 24-well plates (BD Biosciences, San Jose, CA, United States) as described (Garcia-Miguel et al., 2018). Cells were stimulated with IL-6 (300 ng/mL) or PDGF-BB (20 ng/mL), 20,000 cells in 100 μL of DMEM medium without FBS. Results were expressed as the number of migrated cells per photographed field.

A7r5 VSMC proliferation was assessed using MTT assay as previously described (Garcia-Miguel et al., 2018) and by cyclin D1 protein content determined by western blotting.

### Western Blot

Cell cultures were washed with PBS twice and lysed with RIPA lysis buffer (Tris-HCl 10 mM, EDTA 5 mM, NaCl 50 mM, 1% deoxycholic acid and 1% Triton X-100, pH 7.4) containing protease and phosphatase inhibitors (Roche, Indianapolis, IN, United States). The cell lysates were sonicated and centrifuged at 12,000 × *g* at 4°C for 12 min, and the supernatants were collected. Protein concentration of each sample was determined using a BCA Protein Assay Kit. Proteins samples (20–30 μg) were separated by SDS-PAGE 10%, transferred to PVDF membranes and blocked with 5% skim non-fat milk. The membranes were incubated with dilutions of phosphorylated FAK (1:1000, CST #8556, Cell Signaling Technology, Danvers, MA 01923, United States), total FAK (1:1000, SC-1688, Santa Cruz Biotechnology, Dallas, TX, United States), α-smooth muscle actin (α-SMA) (1:20,000, #ab7817, Abcam Cambridge, MA, United States), calponin (1:5,000, #ab78491, Abcam Cambridge, MA, United States), SM22 (1:5,000, #ab14106, Abcam Cambridge, MA, United States); cyclin D1 (1:1000, #ab134175, Abcam Cambridge, MA, United States); collagen type I (1:2,000, cat #ABT123, Merck Millipore, Darmstadt, Germany); β-tubulin (1:5,000, #T8328, Sigma Chem St Louis, MI, United States) and GAPDH (1:10,000, #8795, Sigma Chem St Louis, MI, United States). Membranes were then washed with Tris-buffered saline and Tween 20 (TBS-T) three times, incubated with horseradish peroxidase-conjugated secondary antibodies 1:5000 (mouse. #402335 and rabbit #401315, Millipore Sigma, Burlington, MA, United States) for 1 h and revealed with ECL. Band intensity was determined using a digital imaging system (Syngene, Frederick, MD, United States). Bands were quantified by densitometry using UN-SCAN-IT gel software (Silk Scientific, Inc., Orem, UT, United States).

### Statistical Analysis

Each variable was assessed for normal distribution with the Kolmogorov–Smirnov test. *In vivo* continuous variables are expressed as mean ± standard deviation (SD). Categorical variables are expressed as total number (percentage). Correlations were obtained using Pearson’s analysis. *In vitro* data are presented as mean ± standard error of the mean (SEM) of the indicated number of independent experiments. Data were analyzed using Student’s *t*-test for direct comparisons, one-way ANOVA followed by *post hoc* Holm-Sidak *t*-test, or two-way ANOVA followed by *post hoc* Sidak test for multiple comparisons, according to the experiment. A value of *p* < 0.05 was defined as statistically significant. Outlier analysis was performed using the ROUT method as stated in the legend of the figures. All statistical analyses were performed using GraphPad Prism.

## Results

### Population Characteristics

The athlete groups were composed of 44 male marathon runners, average age 37.5 ± 5.9 years. Athletes were divided into two groups of 22 subjects each, as described in Methods: high-training group (HT, ≥100 km by week) and low-training group (LT, <100 km). The HT group had a training record of 8.1 ± 4.7 years, and the LT group had a training record of 7.3 ± 5.3 years (*p* = 0.61). For comparison, 21 sedentary healthy males were recruited as controls (sedentary group). Both athlete groups had significantly lower cardiac frequency than the sedentary individuals (HT: 55.0 ± 6.1 and LT: 52.0 ± 7.8 vs. sedentary: 68.4 ± 8.3 bpm; *p* < 0.001). Both athlete groups also had a significantly lower bodyweight than the sedentary group (HT: 67.6 ± 8.0 and LT: 73.8 ± 7.2 vs. sedentary: 79.8 ± 8.2 kg; *p* < 0.001 and *p* < 0.05, respectively). The HT group also had a lower body surface area than the sedentary individuals (1.8 ± 0.1 vs. 2.0 ± 0.1 m^2^, *p* < 0.001). The HT group ran the marathon significantly faster than the LT group (197.3 ± 34.5 vs. 227.7 ± 40.7 min, *p* < 0.01). Other demographic characteristics are presented in Table 1.

**TABLE 1:** Participant’s characteristics.

	Groups				
Variable	CT (*n* = 21)	LT (*n* = 22)	HT (*n* = 22)	*p*-value
Age (years)	33.9 ± 4.8	39.3 ± 5.3^4^	36.0 ± 6.2	0.017^1^
Height (cm)	175.5 ± 6.9	174.4 ± 5.4	173.4 ± 7.4	0.598^1^
Weight (kg)	79.8 ± 8.2	73.8 ± 7.2^4,7^	67.6 ± 8.0^6^	<0.0001^1^
Body surface area (m^2^)^3^	2.0 ± 0.1	1.9 ± 0.1	1.8 ± 0.1^5^	0.001^1^
Aortic root diameter (mm)	26.4 ± 5.7	29.7 ± 4.8	28.6 ± 4.1	0.297^1^
Ascending aorta diameter (mm)	30.9 ± 3.5	30.9 ± 2.9	29.0 ± 2.7	0.078^1^
Rest cardiac frequency (bpm)	68.4 ± 8.3	55.0 ± 6.1^6^	52.0 ± 7.8^6^	<0.0001^1^
Marathon time (min)		227.7 ± 40.7	197.3 ± 34.5	0.612^2^

*All values are mean ± SD. CT = control sedentary group; LT = low-training athletes (< 100 km/week); HT = high-training athletes (≥ 100 km/week). ^1^Data were analyzed by one-way ANOVA. ^2^Data were analyzed by unpaired *t*-Test. ^3^Body surface area was calculated using the Du Bois formula (BSA = 0.007184 × Weight^0^ × Height^0^) (Du Bois and Du Bois, 1989) *Post hoc* was performed by Holm-Sidak’s multiple comparisons test. ^4^*p* < 0.05 vs. sedentary; ^5^*p* < 0.001 vs. sedentary; ^6^*p* < 0.0001 vs. sedentary; ^7^*p* < 0.05 vs. Low group.*

### Changes of *Plasma* IL-6 and sIL-6R *Levels* in High-Training (HL) and Low-Training Athletes

**FIGURE 1:**
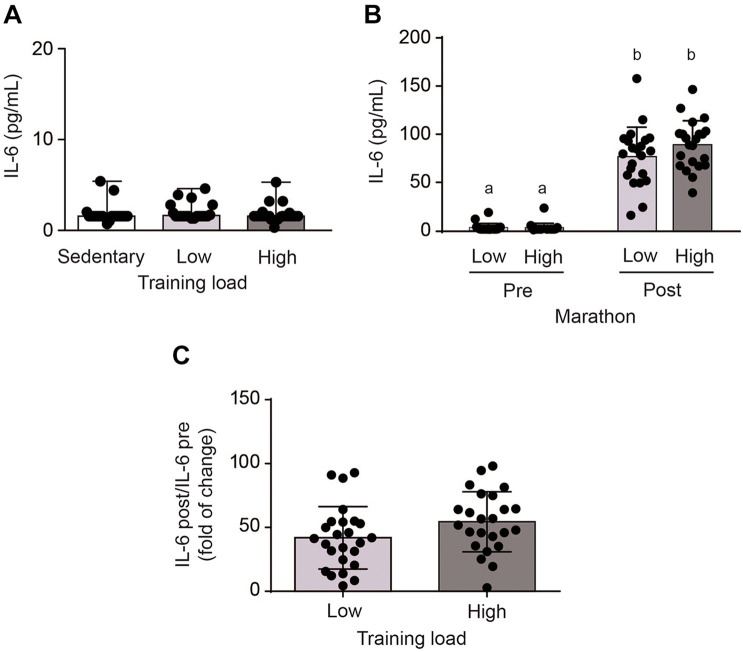
Plasma interleukin-6 (IL-6) levels in sedentary subjects and athletes with high (HT) and low (LT) training loads. HT-group: ≥100 km by week, *n* = 22; LT group: <100 km by week, *n* = 22; and a healthy and inactive physically group (control, CT; *n* = 21). **(A)** Basal venous blood samples were obtained 1 week prior to the marathon. After performing an outlier analysis using the ROUT method (*Q* = 10%), two values in the LT group (corresponding to 11.3 and 18.8) and 1 value in the HT group (corresponding to 23.5) were discarded. Data were analyzed by one-way ANOVA followed by *post hoc* Holm-Sidak’s multiple comparisons test. **(B)** Post-exercise venous blood samples were obtained at the finish line within 5 min of the end of the marathon. Different letters indicate significant differences from different groups at the same time. Data were analyzed by two-way ANOVA followed by *post hoc* Sidak test; a vs. b *p* < 0.0001. **(C)** Changes in plasma IL-6 levels. After performing an outlier analysis using the ROUT method (*Q* = 10%), a single value in the HT group (corresponding to 316) was discarded. Data were analyzed by Student *t*-test. Plasma IL-6 levels were determined using an ELISA kit. All data are presented as mean ± SD.

Basal IL-6 levels were similar in both athlete groups, at values comparable to the sedentary group (Figure 1A). However, plasma IL-6 levels increased significantly immediately after the marathon, from 2.8 ± 4.7 to 89.0 ± 25.1 pg/mL in the HT group and 3.3 ± 4.2 to 76.9 ± 30.6 pg/mL in the LT group (*p* < 0.0001) (Figure 1B). HT and LT groups showed no statistical differences in fold increase changes in plasma IL-6 (49.4 ± 20.3 vs. 37.2 ± 20.5, *p* = 0.056) (Figure 1C). Moreover, no correlation was found between intensity of the exercise, assessed as time of finishing the marathon, and the plasma levels of IL-6 in HT and LT groups. These results suggest that running a marathon increased plasma IL-6 levels, with no differences in the training load.

**FIGURE 2:**
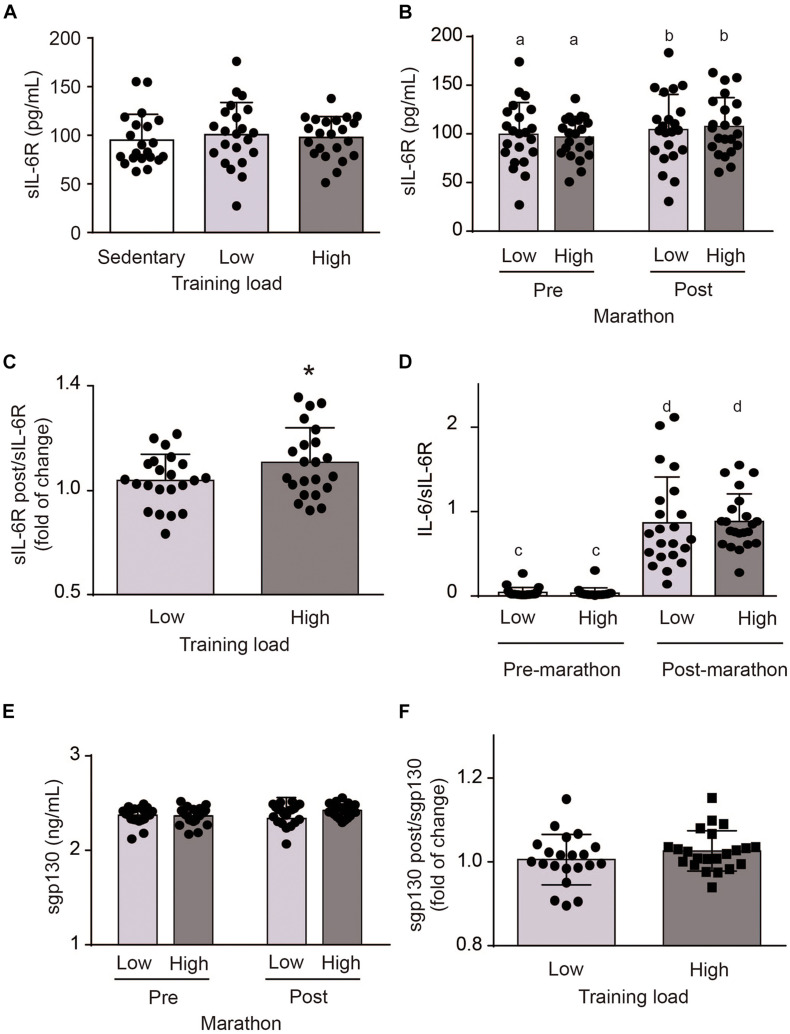
Plasma soluble interleukin-6 receptor (sIL-6R) levels in sedentary subjects and athletes with high (HT) and low (LT) training loads. HT-group: ≥100 km by week, *n* = 22; LT group: <100 km by week, *n* = 22; and a healthy and inactive physically group (control, CT; *n* = 21). **(A)** Basal venous blood samples were obtained 1 week prior to the marathon. Data were analyzed by one-way ANOVA followed by *post hoc* Holm-Sidak’s multiple comparisons test. **(B)** Post-exercise venous blood samples were obtained at the finish line within 5 min of the end of the marathon. Different letters indicate significant differences from different groups at the same time. Data were analyzed by two-way ANOVA followed by *post hoc* Sidak test; a vs. b *p* < 0.05. **(C)** Change in plasma sIL-6R levels. Data were analyzed by Student *t*-test. *p < 0.05 vs. LT group. Plasma sIL-6R levels were determined using an ELISA kit. **(D)** IL-6/sIL-6R ratios were calculated. Different letters indicate significant differences from different groups at the same time. Data were analyzed by two-way ANOVA followed by *post hoc* Sidak test; c vs. d *p* < 0.0001. **(E)** spg130 was determined in the same plasma samples obtained prior to and immediately after the marathon using an ELISA kit. Data were analyzed by two-way ANOVA followed by *post hoc* Sidak test. **(F)** Change in plasma sgp130 levels. Data were analyzed by Student *t*-test. Data are mean ± SD.

Basal sIL-6R levels were also similar in all three groups (Figure 2A). After the marathon, plasma sIL-6R levels were higher in the HT than LT group (106.0 ± 32.7 vs. 98.2 ± 27.2 pg/mL, *p* < 0.01) (Figure 2B). Fractional changes indicated that athletes in the HT group showed a slightly greater increase in plasma sIL-6R levels than the LT (1.12 ± 0.19 vs. 1.05 ± 0.12, *p* < 0.05) (Figure 2C). These results suggest that running the marathon increased plasma sIL-6R levels in a training-dependent manner, with a greater effect in athletes with greater training load.

The increase in plasma IL-6 after strenuous exercise was greater than the increase in plasma sIL-6R. Because IL-6 binds to sIL-6R, we evaluated the proportion of IL-6 that was complexed by sIL-6R at baseline and after the marathon. Under basal conditions the IL-6/sIL-6R ratios in the LT, and HT groups were 0.040 ± 0.059 and 0.033 ± 0.061, respectively, without differences among groups (Figure 2D). The IL-6/sIL-6R ratios in the LT and HT groups increased after the marathon, to 0.87 ± 0.54 and 0.88 ± 0.33 (*p* = 0.91), respectively (Figure 2D). Taken together, these results suggest that at baseline, most of the IL-6 was likely complexed by sIL-6R, while a large proportion of the sIL-6R remained free. However, after the marathon, we observed a greater increase in IL-6 than sIL-6R. Post-exercise, levels of IL-6 and sIL-6R were similar with little free sIL-6R remaining.

The IL-6/sIL-6R signaling is also regulated by soluble gp130 (sgp130) (Taga and Kishimoto, 1997). Basal sgp130 levels were similar in HT and LT groups (2.36 ± 0.09 and 2.37 ± 0.09 ng/mL, respectively, *p* = 0.75) (Figure 2E). Running the marathon did not change the plasma sgp130 levels in HT and LT groups (2.42 ± 0.07 and 2.34 ± 0.22 ng/mL, *p* = 0.09, respectively) (Figure 2E). Fold increase changes indicated that HT and LT group also showed no variations in sgp130 plasma levels (1.03 ± 0.05 vs. 0.99 ± 0.10, *p* = 0.12) (Figure 2F). These results suggest that running the marathon did not modify spg130 in HT and LT groups.

### Changes on Aortic Diameter in High-Training and Low-Training Athletes

**FIGURE 3:**
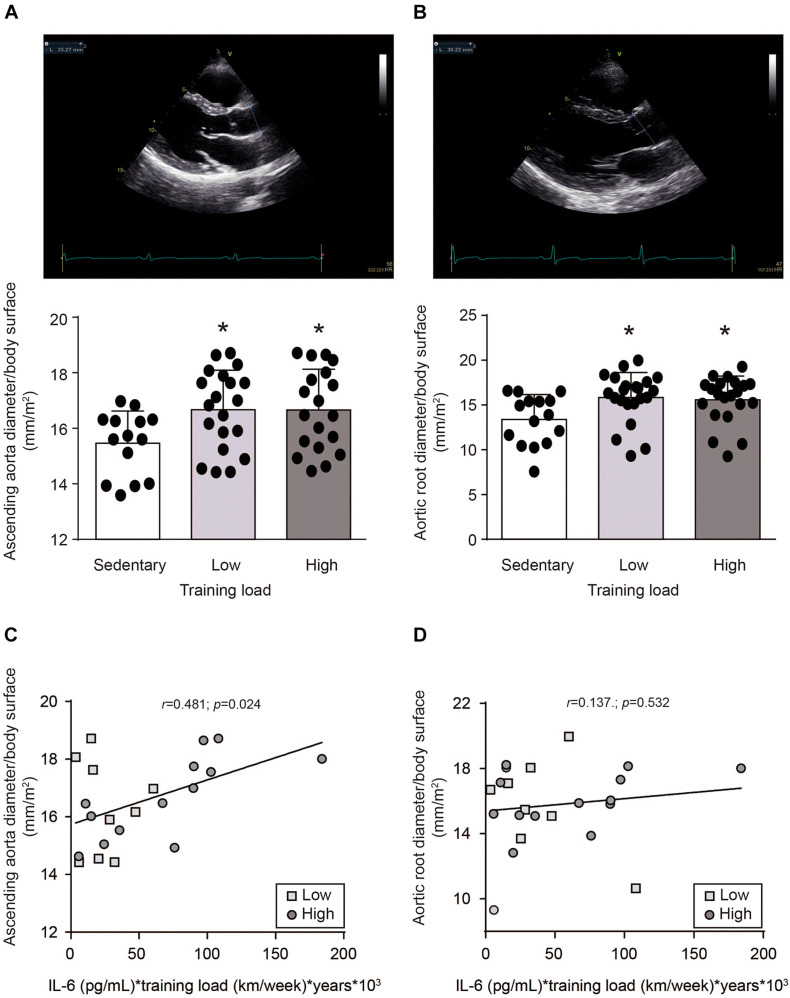
Aortic diameter in in sedentary subjects and athletes with high and low training loads. HT-group: ≥ 100 km by week, *n* = 19; LT group: <100 km by week, *n* = 20; and a healthy and inactive physically group (control, CT; *n* = 14). **(A)** Ascending aortic and **(B)** aortic root diameters were determined by transthoracic echocardiography and normalized by body surface. Upper panels are representative images of the echocardiography. Lower panels are the quantifications. Data are mean ± SD. Data were analyzed by one-way ANOVA followed by Holm-Sidak’s multiple comparisons test. *p < 0.05 vs. sedentary group. Correlation between **(C)** ascending aortic or **(D)** aortic root diameters with plasma IL-6 levels, were evaluated by Pearson’s analysis. Plasma IL-6 levels, determined immediately after running the marathon, were multiplied by training load -considered as kilometers ran per week-, and years of training (□) HT group: (○) LT group.

Arterial remodeling was observed in the athletes. Ascending aortic diameter normalized by body surface area was higher in athletes (HT and LT) than the CT group (16.7 ± 1.4 vs. 15.5 ± 1.2 mm/m^2^, *p* > 0.05) (Figure 3A). This difference was even more pronounced for aortic root diameter (16.0 ± 2.4 vs. 13.4 ± 2.8 mm/m^2^, *p* > 0.05) (Figure 3B). No significant difference in ascending aortic (16.7 ± 1.5 vs. 16.7 ± 1.4 mm/m^2^, *p* > 0.05) or aortic root diameter (16.2 ± 1.9 vs. 15.8 ± 2.8 mm/m^2^, *p* > 0.05) between athletes with different training loads was observed (Figures 3A,B).

To assess whether IL-6 induces vascular remodeling, correlation analysis between aorta diameter and plasma IL-6 levels were performed. However, no correlations were found between exercise-induced plasma IL-6 levels and ascending aortic (Pearson *r* = 0.022; *p* = 0.893) or aortic root (Pearson *r* = -0.060; *p* = 0.704) diameters. Because the increase of plasma IL-6 level induced by exercise is transient (Fischer et al., 2004; Robson-Ansley et al., 2009), we though to evaluate whether the exercise-dependent repetitive increase in plasma IL-6 levels could be responsible for vascular remodeling. Therefore, to assess this parameter, the plasma IL-6 levels evaluated after the race was multiplied by the training load (considered as km/week) and by the years of training. A positive correlation was found between ascending aortic diameter and the plasma IL-6 level weighed by training load (Pearson *r* = 0.481; *p* = 0.024) (Figure 3C). This correlation was not observed with aortic root diameter (Pearson *r* = 0.137; *p* = 0.532) (Figure 3D). Moreover, no correlation was found between ascending aortic diameter and the plasma IL-6 level multiplied only by the training load (Pearson *r* = 0.003; *p* = 0.99) or multiplied only by years of training (Pearson *r* = 0.017; *p* = 0.927), or between ascending aortic diameter and the training load multiplied by the years of training (Pearson *r* = 0.176; *p* = 0.421). These data suggest that repetitive exercise-induced increases of plasma IL-6 levels could be responsible for ascending aortic dilation.

### Effects of sIL-6R on IL-6 and PDGF-BB-Induced Vascular Smooth Muscle Cell Migration

**FIGURE 4:**
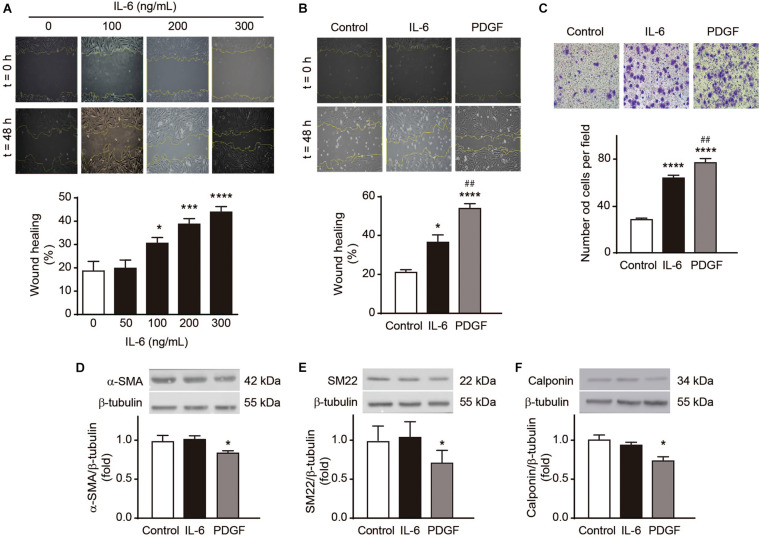
Interleukin-6 (IL-6) induces A7r5 vascular smooth muscle cell migration. **(A)** A7r5 VSMCs were treated with 0, 100, 200, and 300 ng/mL IL-6, and cell migration was assessed by wound healing assay. **(B,C)** A7r5 VSMCs were treated with 300 ng/mL IL-6 or 20 ng/mL PDGF-BB, and cell migration was assessed by wound healing assay **(B)** or Transwell assay **(C)**. Wound healing (%) after 24 h and migrated cells per field in Transwell chamber after 4 h were measured using ImageJ software (*n* = 5). **(D–F)** A7r5 VSMCs were treated with 300 ng/mL IL-6 or 20 ng/mL PDGF-BB for 24 h. α-Smooth muscle actin (α-SMA) **(D)**, SM22 **(E)**, and calponin **(F)** levels were determined by Western blot (*n* = 5). β-tubulin was used as a loading control. Upper panels are representative images. Lower panels are measurements. Data represent mean ± SEM and were analyzed using one-way ANOVA followed by Holm-Sidak’s multiple comparisons test. *p < 0.05, ***p < 0.001, ****p < 0.0001 vs. control; ^##^*p* < 0.01 vs. IL-6.

Phenotypic changes of VSMCs have be usually used as hallmarks of vascular remodeling (Shi et al., 2019). Therefore, to explore how exercise-induced IL-6 and sIL-6R may regulate aortic dilation, we studied the *in vitro* effects of IL-6 and sIL-6R on VSMC phenotype. A7r5 VSMCs were treated with increasing doses of IL-6 (100, 200, and 300 ng/mL), and cell migration was evaluated by wound closure and Transwell migration assay. IL-6 induced cell migration in a dose-dependent manner, with significant differences detectable at doses of 100 ng/mL o more (Figure 4A). However, IL-6 300 mg/mL induced significantly less cell migration than PDGF-BB 20 ng/mL, a well-known inducer of VSMC migration (Abedi and Zachary, 1995), as measured by wound closure (Figure 4B) and Transwell assay (Figure 4C). Moreover, PDGF-BB 20 ng/mL but not IL-6 300 mg/mL, reduced the levels of the contractile proteins α-SMA, SM22 and calponin (Figures 4D–F). These results suggest that IL-6 triggered cell migration, but without modification of the contractile protein content in VSMC.

**FIGURE 5:**
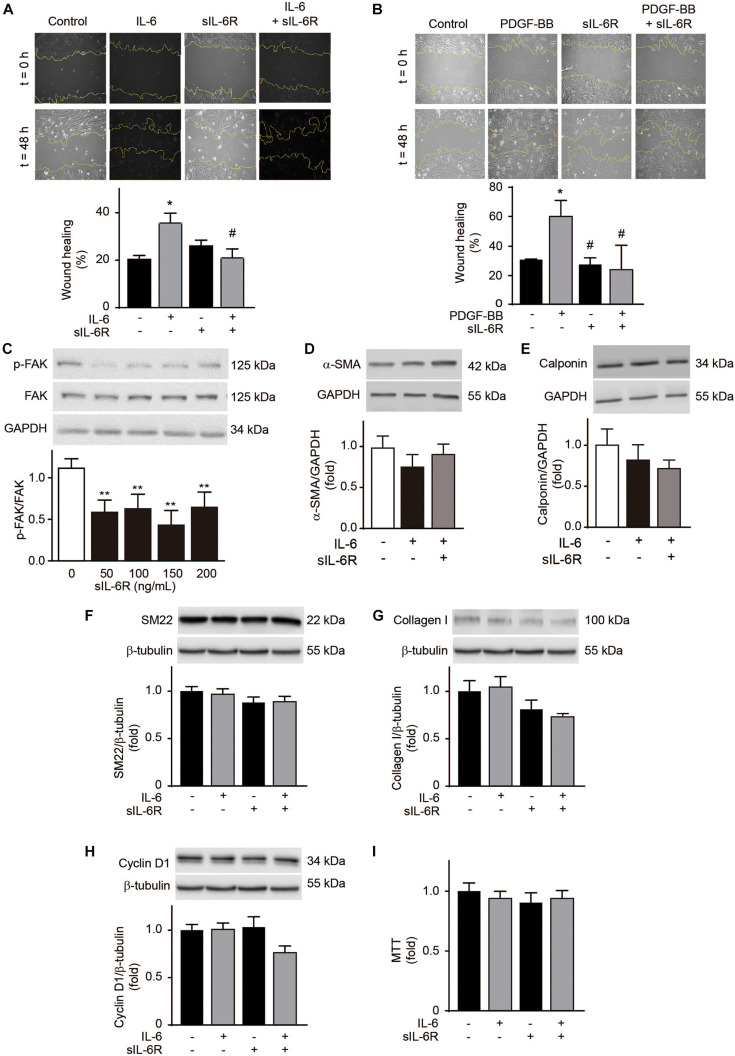
Soluble interleukin-6 receptor (sIL-6R) inhibits interleukin-6 (IL-6)-induced A7r5 vascular smooth muscle cell migration. **(A)** A7r5 VSMCs were treated with 300 ng/mL IL-6, 300 ng/mL sIL-6R, or 300 ng/mL IL-6 + 300 ng/mL sIL-6R. **(B)** A7r5 VSMCs were treated with 20 ng/mL PDGF-BB, 300 ng/mL sIL-6R, or 20 ng/mL PDGF-BB + 300 ng/mL sIL-6R. Wound healing (%) after 24 h was measured using ImageJ software (*n* = 5). **(C)** A7r5 VSMCs were treated with 300 ng/mL sIL-6R for 1 h. Phosphorylated focal adhesion kinase (p-FAK) and total FAK were determined by Western blotting. GAPDH was used as a loading control. **(D–I)** A7r5 VSMCs were treated with 300 ng/mL IL-6, 300 ng/mL sIL-6R, or 300 ng/mL IL-6 + 300 ng/mL sIL-6R for 24 h. α-smooth muscle actin (α-SMA) **(D)**, calponin levels **(E)**, SM22 **(F)**, collagen type I **(G)**, and cyclin D1 **(H)** were determined by Western blot. GAPDH and β-tubulin were used as a loading control. Upper panels are representative images. Lower panels are measurements (*n* = 4). **(I)** Cell proliferation was measured using the MTT assay (*n* = 4). Data represent mean ± SEM and were analyzed using one-way ANOVA followed by Holm-Sidak’s multiple comparisons test in panels **(C–E)**; and two-way ANOVA followed by *post hoc* Sidak test in panels **(A,B,F–I)**. *p < 0.05, **p < 0.01 vs. control; ^#^*p* < 0.05 vs. PDGF-BB.

Because an increase in sIL-6R levels was also observed in the athletes, we evaluated the effects of sIL-6R on VSMC migration. sIL-6R did not alter A7r5 VSMC migration levels (Figures 5A,B). However, sIL-6R completely inhibited IL-6-induced cell migration (Figure 5A). To assess whether this inhibition was an IL-6 specific or general effect, we measured the effect of sIL-6R on PDGF-BB-induced cell migration. The same inhibition of cell migration was observed in A7r5 VSMCs stimulated with PDGF-BB (Figure 5B). Cell migration is a complex process involving a series of steps. In early stages, focal adhesions, required for cell migration, are formed through increased focal adhesion kinase (FAK) phosphorylation (Behmoaram et al., 2008). sIL-6R decreases p-FAK levels in a dose-dependent manner (Figure 5C). These results suggest that sIL-6R inhibits both IL-6- and PDGF-BB-induced A7r5 VSMC migration and that this inhibition occurs in the early stages of the cell migration process. Furthermore, sIL-6 did not modify the levels of the contractile proteins α-SMA, calponin and SM22 in VSMC treated or non-treated with IL-6 (Figures 5D–F). Likewise, IL-6 did not increase collagen type I content in A7r5 VSMCs and this content was not modified by sIL-6R treatment (Figure 5G). Finally, IL-6 and IL-6 + sIL-6R did not alter A7r5 VSMC proliferation measured by tetrazolium blue reduction assay and cyclin D1 content (Figures 5H,I). These results suggest that IL-6 only induces A7r5 VSMC migration without altering other phenotypic markers and sIL-6R inhibits IL-6-induced cell migration.

## Discussion

In this work we showed that strenuous exercise, such as a marathon, increases plasma IL-6 and sIL6-R levels. Epidemiological studies have described a negative association between amount of regular physical activity and basal plasma IL-6 levels (Pitsavos et al., 2005). A similar reduction in basal IL-6 was found in obese individuals subjected to a hypocaloric diet and regular aerobic exercise (Kohut et al., 2006) and in patients with coronary artery disease after training (Goldhammer et al., 2005). Aerobic training for 10 months also decreased basal plasma IL-6 in >64 years old adults (Kohut et al., 2006). However, in other studies have reported that training had no effect on basal IL-6 levels (Leggate et al., 2012; Isaksen et al., 2019). In this study, we observed no change in basal IL-6 levels in response to training. This result is likely due the use of young healthy individuals (< 50 years of age) with low basal inflammation levels subjected to an extremely long training period (> 5 years).

In terms of sIL-6R, a 12-week physical training program reduced basal plasma levels in 24 patients with stable congestive heart failure (Adamopoulos et al., 2002). Moreover, a hypocaloric diet and exercise reduced basal plasma sIL-6R in 17 obese postmenopausal women (You et al., 2004). A comparable decrease was observed in 12 obese males subjected to 2 weeks of high-intensity intermittent training (Leggate et al., 2012). Our data show no differences in basal sIL6R among the athletes and inactive physically but healthy control group. This disagreement could be due to the participation of young, healthy, and well-trained subjects, as mentioned above.

IL-6 levels increased after running a marathon in the athletes we studied, as expected, given that a pronounced increase in plasma IL-6 has been consistently reported after intense weight-bearing exercise such as running, which involves several large muscle groups (Catoire and Kersten, 2015). Few studies have evaluated the effect of training on exercise-induced elevation in plasma IL-6. One of the available studies assessed the effects of a 10-week program consisting of 1 h of knee-extensor exercises 5 times per week. In that study, a group of 7 healthy men showed a less marked post-exercise elevation in skeletal muscle IL-6 mRNA after the training program, but no change in post-exercise plasma IL-6 elevation (Fischer et al., 2004). In our work, the HT group ran the marathon faster than the LT, suggesting a greater workload that could explain the higher increase in plasma IL-6. However, we do not detect higher IL-6 increases in HT athletes than LT athletes and we also did not find association between time of finishing the marathon and plasma levels of IL-6. In this context, even that LT and HT runners have different workload, our data suggest that in these athletes, probably by the high demanding exercise of running a marathon, this difference has little or no impact in the increase of IL-6.

Similarly, running the marathon increased plasma sIL-6R levels. This result is consistent with a study of 13 trained male individuals that cycled a total of 468 km over 6 days. The group showed significantly elevated plasma sIL-6R at rest over the duration of the event compared with the pre-event baseline (Robson-Ansley et al., 2009). Our HT group also showed a greater increase in sIL-6R levels after running a marathon. To date, no other studies have reported whether different training loads affect exercise-induced increases in sIL-6R.

The sgp130 interacts with the IL−6/sIL−6R complex but not with IL−6 alone (Jostock et al., 2001). It was proposed that sgp130 selectively capture the IL-6/sIL-6R complex, thus inhibiting the activity of this complex (Demyanets et al., 2012; Wolf et al., 2016). Gray et al. (2008) described an increase in sgp130 plasma levels in 12 healthy subjects submitted to a submaximal exercise bout of cycling up to volitional exhaustion. This effect was not observed with less intense exercise (Patterson et al., 2008). We also did not observed changes in sgp130 plasma levels after running the marathon, neither in HT nor in LT groups.

Several mechanisms have been proposed to be responsible for inducing changes in arterial wall thickness in response to exercise training. These mechanisms include shear stress, blood pressure, vascular tones, oxidative stress, sympathetic nervous system, and inflammation (Newcomer et al., 2011; Thijssen et al., 2012; Green and Smith, 2018). However, the role of myokines in the exercise-induced vascular remodeling have been poorly studied. Here we described that IL-6 and sIL-6R can also be involved in the exercise-induced vascular remodeling. Although, our *in vivo* studies are only observational, some hints can be obtained by evaluating the *in vitro* effects of IL-6 and sIL-6R on cultured VSMC.

We observed that both athlete groups showed a larger ascending aortic and aortic root diameter than the sedentary group. Repeated episodes of exercise can produce a structural adaptation in the vessels known as physiological vascular remodeling, which involves arterial enlargement without fibrosis or immune cell infiltration (Green and Smith, 2018). Accordingly, we observed a positive correlation between ascending aorta diameter with plasma IL-6 levels, only when training load and years of training were considered. This result suggests that a repetitive and long-lasting increase of plasma IL-6 levels could be responsible for ascending aorta dilation induced by exercise. However, this conclusion should be taken with caution. During resistance exercise a lower IL-6 increase than endurance exercise is detected (Calle and Fernandez, 2010). However, it is described that resistance exercise causes more aortic enlargement than does endurance exercise (Stephen Hedley and Phelan, 2017). In that context, other myokines have been described to be increased after running a marathon. Increases in the levels of myostatin, irisin, sclerostin, osteoprotegerin, IL-8 and IL-10 have been described (Santos et al., 2020; Sliwicka et al., 2021). Moreover, other myokines, such as IL-8 and IL-15, are also increased after exercise (Pedersen and Febbraio, 2008). Hence, other factors, besides IL-6, could be also involved in the artery remodeling triggered by exercise. Therefore, in this context, our data only provides indirect evidence that IL-6 could be involved in exercise-dependent vascular remodeling.

An increased arterial diameter due to arteriogenesis is characterized by a phenotypic switch in VSMCs from a contractile to a migratory and proliferative state (Cui et al., 2009; Poling et al., 2011). Chronic exposure to flow changes and shear stress has been reported to lead to thinning of the carotid artery wall and an increase in VSMC proliferation (Green et al., 2017). In addition, exercise-induced remodeling stimulates the release of growth factors such as PDGF, which has been shown to induce VSMC migration and proliferation (Green et al., 2017). Induction of VSMC migration, a main feature of arteriogenesis, is triggered by IL-6. In this work, we demonstrate that IL-6 induces A7r5 VSMC migration in a dose-dependent manner. This result also agrees with previous observations that IL-6 induces VSMC proliferation and migration (Morimoto et al., 1991; Wang and Newman, 2003). Moreover, we observed that IL-6 did not reduce the contractile levels of A7r5 cells, did not induced cell proliferation and did not increase collagen type I content. This data suggest that IL-6 increase cell migration without modification of the other phenotypic markers characteristic of VSMC dedifferentiation, i.econtractile proteins, cell proliferation and collagen type I synthesis. This effect of IL-6 could be relevant, because during exercise induced vascular remodeling artery structure was modified without alteration in vascular contractility (Thijssen et al., 2012). Moreover, exercise training is related to an outward remodeling of the arterial lumen and a decrease in wall thickness while in cardiovascular diseases an arterial wall thickening is observed (Thijssen et al., 2012). Therefore, these data suggest that a different phenotypic change should occurs in VSMC in both contexts. Our data shows que IL-6 only increased VSMC migration without modifying contractile proteins, collagen type I and cell proliferation. This phenotypic change is different to those observed associated with cardiovascular diseases (Shi et al., 2019). Because the HT group showed a greater increase in IL-6, and likely experienced greater shear stress due to the greater training load, aortic remodeling was also likely increased. Previous studies have reported that elite athletes, who exhibit increased conduit vessel diameter at rest, experience further structural vascular adaptations due to intense training (Naylor et al., 2006). Surprisingly, we found that the HT athletes had an aortic diameter similar to that of the LT group. This observation suggests a compensatory or regulatory mechanism that could limit the vascular remodeling induced by exercise.

IL-6 binds to the plasma membrane-associated IL-6 receptor (IL-6R). The IL-6/IL-6R complex then associates with gp130, inducing homodimerization and initiation of signaling (Taga and Kishimoto, 1997). A soluble form of IL-6R (sIL-6R) has also been described in various body fluids, including blood (Demyanets et al., 2012). This sIL-6R is preferentially produced by proteolysis of the membrane IL-6R (Taga and Kishimoto, 1997). The sIL-6R regulates the IL-6 actions (Taga and Kishimoto, 1997). Here we found that sIL-6R treatment inhibited both IL-6- and PDGF-induced migration in A7r5 VSMCs. In addition, sIL-6R reduced FAK phosphorylation, a parameter used to assess the early stages of cell migration. In fact, FAK phosphorylation is required for VSMC migration induced by IL-6 (Wang and Newman, 2003). IL-6 triggers FAK phosphorylation through an actin polymerization (Wang and Newman, 2003) and STAT3 dependent mechanism (Wei et al., 2018). Therefore, the sIL-6R released after high-intensity exercise could inhibit the IL-6- and PDGF-induced migratory response of VSMCs and consequently attenuate physiological vascular remodeling in more highly trained athletes. However, additional experiments are required to clarify this hypothesis.

## Study Limitations

The primary study limitations were: (a) Study groups were small due to the challenging enrollment process and technical complexity of taking samples at the marathon finish line. (b) We did not study any female athletes (who may have a different adaptation process). (c) The impossibility of performing interventions to manipulate IL-6/sIL-6R levels in athletes limits the scope of our research. (d) Our data is only valid in male, caucasian, marathon athletes and cannot be extrapolated to other sport disciplines. (e) Results are obtained only in marathon runners and cannot be generalized to other type of exercises or sports. (f) We only assessed IL-6, sIL-6R, and sgp130 and the involvement of other myokines in the aorta remodeling induced by exercise cannot be ruled out.

## Data Availability Statement

The raw data supporting the conclusions of this article will be made available by the authors, without undue reservation.

## Ethics Statement

The studies involving human participants were reviewed and approved by Ethics Committee of Pontificia Universidad Católica de Chile, in observance of the Declaration of Helsinki on experimentation in humans’ beings (project No 16082603). The patients/participants provided their written informed consent to participate in this study.

## Author Contributions

LoG, MO, RT, and MC contributed conception and design of the study. PV-F, AP, TH-D, IN-S, NC-A, FS-O, FC-B, JM, and NB performed the experiments. PV-F, TH-D, and MC performed the statistical analysis. PV-F and MC wrote the first draft of the manuscript. RT and LuG wrote sections of the manuscript. All authors contributed to manuscript revision, read, and approved the submitted version.

## Conflict of Interest

The authors declare that the research was conducted in the absence of any commercial or financial relationships that could be construed as a potential conflict of interest.

## Publisher’s Note

All claims expressed in this article are solely those of the authors and do not necessarily represent those of their affiliated organizations, or those of the publisher, the editors and the reviewers. Any product that may be evaluated in this article, or claim that may be made by its manufacturer, is not guaranteed or endorsed by the publisher.
